# Diabetic muscle infarction and diabetic dermopathy two manifestations of uncontrolled prolong diabetes mellitus presenting with severe leg pain and leg skin lesions

**DOI:** 10.1186/2251-6581-13-38

**Published:** 2014-02-21

**Authors:** Saeedeh Shenavandeh, Amir Anushiravani, Mohammad Ali Nazarinia

**Affiliations:** 1 Division of Rheumatology, Department of Internal Medicine, Shiraz University of Medical Sciences, Shiraz, Iran; 2 Departments of Internal Medicine, Student Research Center, Shiraz University of Medical Sciences, Po Box: 71345–1414, Shiraz, Iran

**Keywords:** Diabetic muscle infarct, Diabetic dermopathy, Leg pain

## Abstract

Diabetic muscular infarction (DMI) is a rare manifestation which can be seen in patients with long-standing diabetes mellitus. Patients usually come with painful swelling of an involved muscle in one extremity. MRI and biopsy histology can help diagnose this condition. Diabetic dermopathy is another manifestation of patients with diabetes.

We present a patient with uncontrolled diabetes type 2 presented with pain, swelling, and a palpable tender mass in one leg along with new skin lesions. Biopsy of the skin lesion and T2-weighted MRI of the leg helped differentiate DMI and dermopathy.

## Background

Diabetic muscular infarction (DMI) is a rare condition which has been seen in patients with long-standing diabetes who usually have other complications of poor glycemic control. It has been reported as aseptic myonecrosis, ischemic myonecrosis, and tumoriform focal muscular degeneration.

DMI was first described by Angervall and Stener in 1965 [1]. It presents as an acute onset of painful swelling of the affected muscle. MRI and biopsy histology can help differentiate it with other similar conditions. Even though DMI has been reported for over 45 years, still, less than 200 cases have been reported [1].

We are presenting a rare manifestation of uncontrolled diabetes type 2 in a patient who came with pain, swelling, and a palpable tender mass in one leg along with new skin lesions.

## Case presentation

A 57 year old woman, with uncontrolled diabetes type 2 and hypertension, referred to a university hospital clinic with a complaint of right leg swelling and tenderness since 40 days prior to admission. She didn’t give a history of trauma, bite, muscle weakness or fever. She had received analgesics (ibuprofen 400 mg bid and diclofenac 50 mg bid) and cephalexin without any improvement.

**Figure 1:**
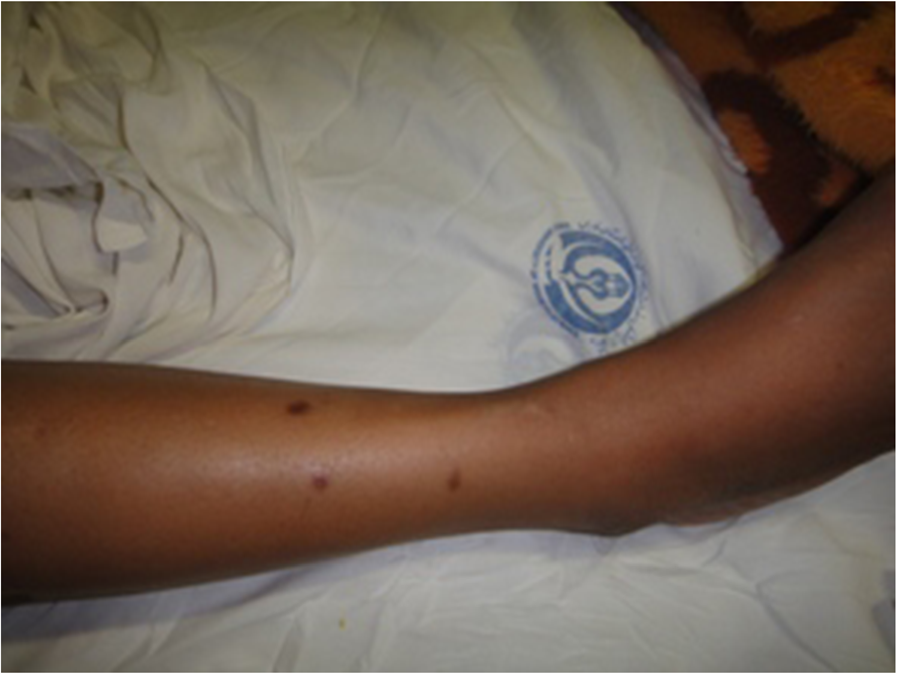
A maculopapular rash in the lower extremitiy representing diabetic dermopathy.

**Figure 2:**
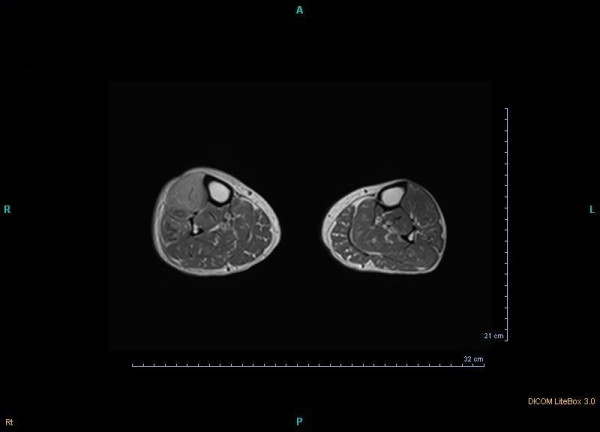
MRI shows a heterogenous enhancement in right tibialis anterior muscle in T2-weighted images.

**Figure 3:**
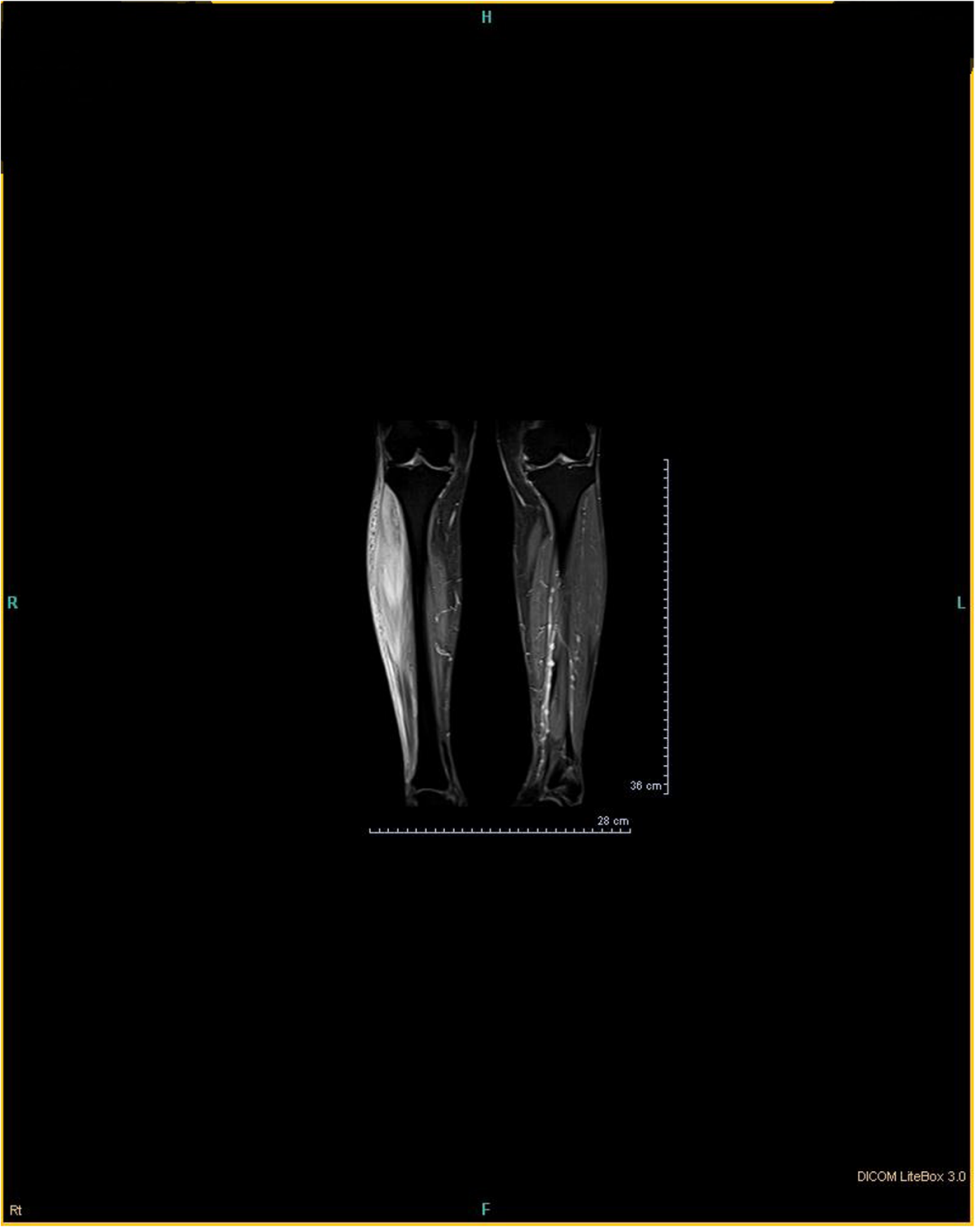
MRI shows a heterogenous enhancement in right tibialis anterior muscle in T2-weighted images.

The patient had uncontrolled diabetes type 2 for over 10 years despite being on a maximum dose of oral hypoglycemic agents. Her blood pressure was controlled with losartan and atenolol. On physical examination she had swelling in her right shin with severe tenderness, 1+ pitting edema and a 0.5 cm size difference compared with her left leg. The pain was so severe that she could not walk and any weight-baring activities caused extreme pain. Proprioception and vibration were impaired in both lower extremities. There were 2-3 round-oval hyperpigmented macules on her right lower extremity about 2 cm in diameter that appeared recently during her problem course without any itching, for which skin biopsy documented diabetic dermopathy. (Figure 1) Color Doppler sonography of her right leg veins was normal. Soft tissue sonography revealed moderate swelling and inflammation with no sign of cellulitis or abscess. MRI showed a heterogenous enhancement in right tibialis anterior muscle in T2-weighted images (Figures 2 and 3). Erythrocyte sedimentation rate (ESR) was 67 mm/hr. Creatine phosphokinase and lactate dehydrogenase was normal. HbA1C was 12.0%. Complete blood count, renal and liver function, lipid profile, calcium, phosphorus and uric acid were also normal.

Our treatment strategy was to control her glucose level with NPH and regular insulin. We also gave her naproxen 500 mg bid with occasional morphine injections. She was advised to avoid weight-bearing. Aspirin, low dose heparin, gabapentine, alprazolam and imipramine were also given. After 2 weeks the patient had a moderate improvement in pain and was able to walk with a walker.

## Conclusions

DMI is an uncommon complication of long-standing uncontrolled diabetes of both type 1 and 2 [2]. The pathogenesis is not clear. The most likely hypothesis is vascular diseases like arteriosclerosis and diabetic microangiopathy [3]. Thigh muscles are the most commonly affected and reports of lower leg involvement are very rare [4]. Upper extremity involvement has also been reported [5].

Clinical features usually consist of local swelling, limitation and pain on motion, tenderness, and a palpable painful mass, usually without fever and severe induration. Muscle enzymes are usually not elevated and an elevated ESR was seen in about 50%.

Diagnosis can be made combining clinical presentation and radiologic imaging. MRI is one of the best methods. Electromyography has been shown to help in some cases [6]. Biopsy confirms the diagnosis in over 90% of cases [7], but since it has potential complications, it should be reserved for atypical cases were diagnosis is hard to make. The main reason why biopsies are not performed regularly is that those who have biopsies also have a longer course of pain and associated problems.

The most common differential diagnoses are deep venous thrombosis and pyomyositis, although soft-tissue abscess, necrotizing fasciitis, dermatomyositis, proliferative myositis, focal myositis, nodular myositis, primary lymphoma of muscle, benign tumors or sarcomas of the muscle, diabetic amyotrophy, osteomyelitis, exertional muscle rupture, and ruptured Baker’s cyst were also noted [8,9].

Diabetic dermopathy, sometimes termed *pigmented pretibial papules*, or “diabetic skin spots,” begins as an erythematous area and evolves into an area of circular hyperpigmentation that is in patients with long-standing diabetes and no treatment is warranted [10].

In our patient the presentation was localized muscle involvement and skin lesions in an uncontrolled diabetic patient, which DMI along with diabetic dermopathy had been diagnosed. This was the first report of DMI and diabetic dermopathy together. As the rate of diabetes mellitus is rising, we might be seeing these patients more often and we should be concerned about them. Treatment is controversial, but trials of anti-platelets, anticoagulation, analgesics, off-loading, rehabilitation methods, and anti-depressants have been used. We recommend more thorough clinical trials to see if these treatment regimens significantly improve the outcome.

In conclusion DMI is an uncommon complication of long-standing uncontrolled diabetes presenting with swelling and severe pain usually in lower extremity and usually prompts an extensive diagnostic workup to find etiology of localized myositis which might be unnecessary if the physician has a high level of suspicion, and a T2-weighted MRI images can be a good diagnostic help.

## Consent

Written informed consent was obtained from the patient for publication of this case report and any accompanying images.

## Competing interest

The authors declare that they have no competing interest.

## Authors’ contributions

AA carried out data gathering and review of literature, participated in the sequence alignment and drafted the manuscript. SS also carried out review of literature, participated in the sequence alignment and drafted the manuscript. MAN participated in the sequence alignment and drafted the manuscript. All authors read and approved the final manuscript.
